# Knowledge of Diabetic Retinopathy among Primary Care Nurses Performing Fundus Photography and Agreement with Ophthalmologists on Screening

**DOI:** 10.3390/nursrep13030093

**Published:** 2023-08-11

**Authors:** Domingo Ángel Fernández-Gutiérrez, Janet Núñez-Marrero, Carlos Enrique Martínez-Alberto, Martín Rodríguez-Álvaro, Retisalud-Tenerife Primary Healthcare Research Group, Alfonso Miguel García-Hernández, Pedro Ruymán Brito-Brito

**Affiliations:** 1 Nursing Department, Faculty of Healthcare Sciences, University of La Laguna, 38200 Santa Cruz de Tenerife, Spain; dfernand@ull.edu.es (D.Á.F.-G.); pbritobr@ull.edu.es (P.R.B.-B.); 2 Primary Care Management Board of Tenerife, The Canary Islands Health Service, 38003 Santa Cruz de Tenerife, Spain; jnunmar@gobiernodecanarias.org; 3 Nuestra Señora de Candelaria School of Nursing, 38010 Santa Cruz de Tenerife, Spain; 4 Health Services Management Board of La Palma, The Canary Islands Health Service, 38713 Breña Alta, Spain

**Keywords:** health knowledge, attitudes, practice, diabetic retinopathy, early diagnosis, nursing, primary health care

## Abstract

Diabetic retinopathy (DR) is one of the complications of diabetes mellitus (DM), with macular oedema being one of the leading causes of avoidable blindness among individuals with DM worldwide. Fundus screening is the only method for early detection and treatment. High-quality training programmes for professionals performing primary care screening are essential to produce high-quality images that facilitate accurate lesion identification. This is a two-phase observational, descriptive, and cross-sectional study. The first phase analysed DR knowledge in a sample of nurses. The second phase explored agreement on DR screening between referral ophthalmologists in image assessment (gold standard) and a small group of nurses involved in the previous phase. In phase 1, the agreement rate for screening results was 90%. In phase 2, the overall raw agreement on the screening of fundus photography results between nurses and ophthalmologists was 75% (Cohen’s kappa = 0.477; *p* < 0.001). Agreement on screening with ophthalmologists was moderate, suggesting that implementing a specific training programme for nurse-led imaging screening would help develop this competence among nurses, ensuring a good level of agreement and patient safety and adding value for users, and also for the sustainability of the healthcare system. This study was not registered.

## 1. Introduction

The World Health Organisation (WHO) describes diabetes mellitus (DM) as a metabolic disorder characterised by the presence of hyperglycaemia when left untreated [1]. There are currently an estimated 541 million people in the world with the disease, and this figure increases to 783 million in projections for 2045 [2]. Diabetic retinopathy (DR) is one of the complications of DM and is often described as a chronic and progressive deterioration of the retinal vessels (microangiopathy) associated with prolonged hyperglycaemia. This condition affects one third of individuals with DM after 15 years of disease progression. Of the 95 million people with DR today, one third experience vision problems and 7.6% experience macular oedema [3], which is one of the leading causes of avoidable blindness in people with DM worldwide [4]. DR remains asymptomatic until damage is already present. Therefore, regular screening is important to initiate treatment as early as possible [5].

According to the principles of population screening, as established by Wilson and Junger in 1968 and adopted by the WHO, screening programmes are not intended to make a diagnosis but to identify individuals suspected of having the disease so they can be referred to the relevant specialists for diagnosis and treatment [6]. In the case of DR, programmes should aim to identify retinal changes, so that treatment can be provided before visual impairment or blindness occurs. The WHO has recommended implementing effective screening programmes in the community setting to prioritise early diagnosis, management, and treatment of this complication. The Saint Vincent Declaration, adopted in Italy in 1989, was the starting point for subsequent agreements, such as those reached in the Liverpool Declaration in 2005. In compliance with these recommendations, all European countries committed themselves to implementing programmes to reach 80% coverage of patients with diabetes, as well as to providing universal access to laser treatment [7].

The gold standard for DR screening is the screening performed by ophthalmologists. However, systematic specialist screening of these patients is resource- and time-intensive and is often not necessary as the patient does not yet have lesions requiring treatment [8]. In addition, the number of ophthalmologists available to attend to people requiring screening is very limited. In European countries, such as Spain, Belgium, Germany, Sweden, Croatia, Hungary, and Slovenia, there are between 5 and 10 ophthalmologists per 100,000 inhabitants [9]. It is well known that if screening were performed outside ophthalmology practices and by other professionals, it would enable specialists to see only users who really need to be screened. As a result, individuals with DR could be seen by ophthalmologists at an earlier stage of the disease, which could increase their chances of receiving laser photocoagulation treatment or intravitreal pharmacological agents, preventing vision loss in cases of proliferative DR or stabilising or improving vision in cases of diabetic macular oedema [10].

It is considered that for the screening method to be acceptable, it must have more than 80% sensitivity for detection. Neither a direct, non-mydriatic ophthalmoscopy performed by a non-ophthalmologist (with 36–63% sensitivity) nor a visual acuity measurement (50% sensitivity) meet this requirement, which is why these methods were discarded [11,12].

Photographic methods such as digital fundus photography using a non-mydriatic fundus camera (with or without pupillary mydriasis) are valid methods, with sensitivity and specificity values above 80% and 90%, respectively [13].

Fundus photography, in conjunction with telemedicine, allows other health professionals available in the community to perform this task, provided they have received specific training and are equipped with the appropriate technology. Non-ophthalmologists who can screen for DR include general practitioners, nurses, and optometrists [14].

In Spain, in line with the Primary Health Care (PHC) model in the community, general practitioners have been proposed as fundus photography interpreters. The results available in the literature indicate that, after proper education and training, these professionals can interpret these images with 85% sensitivity and specificity compared to ophthalmologists [12]. However, the PHC system in Spain is under severe strain regarding the prospect of replacing these medical specialists, as 28% of them are 60 years old or older and 63% are 50 years old or older. This specialty has the poorest forecast in terms of shortages in five years’ time in Spain, with an estimated shortage of approximately 9000 physicians [15]. Screening programmes in the field of ophthalmology are constantly evolving and readjusting to optimise procedures in line with the different requirements of the various healthcare systems. Also, current evidence shows a growing trend in studies that propose incorporating nurses as DR screening interpreters, as they also attain sensitivity and specificity values of over 85% when compared to ophthalmologists [7,16,17].

In the Autonomous Community of the Canary Islands, Spain, a community care programme for DR screening in people with type 2 diabetes called Retisalud was implemented in 2006 as the official DR screening model managed by the PHC teams, which are formed by a physician and a nurse. The project includes the training of physicians as fundus photography interpreters. Physicians receive targeted training that formally qualifies them to assess such images [18,19]. Additionally, in the Tenerife Healthcare Area, nurses must also pass a 50-h, 5.8-credit theoretical, and practical course—accredited by the Health Professions Training Commission—to be able to carry out their healthcare work within the Retisalud programme on fundus photography consultation. This course was designed to train nurses in the various models of fundus cameras available in the department so that they can obtain images with high diagnostic quality and identify fundus structures, as well as the most common lesions associated with DR.

The aim of this article was to describe the level of knowledge of DR among PHC nurses who perform fundus photography in order to analyse the quality of the training received and to assess the degree of agreement on screening for DR between nurses who received that training and ophthalmologists.

## 2. Materials and Methods

### 2.1. Design and Sampling Method

This is an observational, descriptive, cross-sectional study carried out in two phases. In the first phase, knowledge of DR was analysed in a sample of nurses. They were selected using a convenience sampling method from those nurses who had received training on and routinely carry out outpatient fundus photography in PHC in the Tenerife Healthcare Area of the Canary Islands Health Service (SCS). The second phase examined the agreement on DR screening between a group of referral ophthalmologists in the assessment of these images (gold standard) and a small group of nurses participating in the previous phase. For both phases, randomly selected fundus photographs—available from electronic health records (EHRs)—were selected from patients with type 2 DM who had undergone this test in a PHC facility in 2022. In addition, the results of each image should have been recorded in the EHRs as assessed by the referring ophthalmologist at the hospital level. Considering that the aims of the study are to calculate correlations and agreements between nurses and ophthalmologists, as well as measure their level of knowledge on the topic rather than calculating prevalence, the necessary sample sizes were estimated to be at least 65 nurses in phase 1 and approximately 100 fundus photographs of 100 different patients in phase 2. This would facilitate the calculation of non-parametric correlation coefficients with a significance of at least 0.28, thereby giving the study a 90% power in two-tailed hypothesis testing at an alpha significance level of 0.05 and 95% confidence intervals.

### 2.2. Study Setting

In the PHC of the Tenerife Healthcare Area, public healthcare services are provided to individuals with DM and other chronic health problems. Based on EHR data mining, the percentage of the adult population enrolled in a DM care programme is 8.4%. Of these, 71.3% had a glycosylated haemoglobin (HbA1C) value recorded in the previous 16 months, with 20.2% having an HbA1C value of 8 or higher in their last record.

The Tenerife Healthcare Area has 42 basic healthcare districts (Zonas Básicas de Salud, ZBSs) and a total of 103 PHC facilities, where 795 nurses carry out their professional duties. Of these, 165 are trained in the performance of high-quality diagnostic non-mydriatic fundus photography tests. This test is carried out in 39 ZBSs, covering the entire population of the Tenerife Healthcare Area. The training programme that these nurses attended focuses on acquiring theoretical and practical skills in a 50 h B-learning course, which is a prerequisite for performing fundus photography tests on people with DM in the PHC in the Tenerife Healthcare Area. As such, 59.5% of the population with DM had had a fundus photography test performed in PHC in the past two years, with 86.3% being assessed in the following month by at least one of the PHC team professionals, a physician, and/or a nurse, prior to the ophthalmologist’s assessment and diagnosis.

### 2.3. Data Collection Procedure 

Before its inception, the study was approved by the Research Ethics Committee at the Canary Islands University Hospital Complex (Tenerife, Canary Islands, Spain) under the reference code CHUC_2019_39. 

For phase 1 of the study, the research team designed a fundus photograph screening and assessment exercise using Google Forms^®^. The photographs were randomly selected from the Retisalud EHR consultation module and taken in PHC facilities in the Tenerife Healthcare Area from people with DM during the same year. Only images with EHR results recorded after assessment and diagnosis by the referring ophthalmologist were eligible. The knowledge assessment exercise involved the evaluation of a total of 20 images. The link to the online form was sent by corporate email to all eligible nurses who agreed to participate in the study: they must have been previously trained in the performance and assessment of outpatient non-mydriatic fundus photography in PHC, irrespective of the time elapsed since their training. In the knowledge assessment exercise, sociodemographic and professional experience data were collected. In addition, a screening result is to be selected for each image: normal, non-normal, or invalid. If the image is considered non-normal, the type of lesion(s) observed should be specified: hard exudates, soft exudates (cotton wool spots), haemorrhages, and microaneurysms. Finally, participants are asked to determine whether the image is likely to be considered as invalid, no DR, mild DR, moderate DR, or severe DR. Thus, the level of knowledge is measured based on the number of matching results when compared to those recorded by the referring ophthalmologist for each image. Considering that for each fundus photograph the nurses must assess three aspects (screening, lesions observed, and presence/degree of DR), the number of matching answers for each image ranges from 0 to 3, totalling between 0 and 60 matching answers, which determines the overall level of knowledge. The link to these knowledge assessment forms was sent to the participating nurses from July 2022 to October 2022.

For phase 2, fundus photographs were selected from individuals with DM who had undergone testing at a PHC facility and who were assigned to the nurses who had agreed to participate in this second phase. As in the previous phase, photographs were randomly selected from EHRs, and which had a record of the results registered by the referral ophthalmologist. At this stage, sociodemographic and clinical data are collected from patients whose fundus images are included in the study, as well as information relating—for the agreement tests—to two possible outcomes for each fundus photograph: screening (normal versus non-normal) and presence/degree of DR (no DR, mild DR, moderate DR, or severe DR). Should the information on a given EHR not be complete, the case will not be included in the study and new data will be collected from a different patient.

To randomise image selection in both phases, an anonymous list is made by assigning a unique number to each participating nurse in order of inclusion in the study. A fundus image from each nurse’s latest diabetes patients is then retrieved sequentially from each EHR in the Retisalud consultation module until the total number required is reached, i.e., 20 for phase 1 and 100 for phase 2. The fundus photographs selected for phase 1 were taken between May and June 2022, and those selected for phase 2 were taken between August and September 2022.

### 2.4. Variables 

Nurse variables (phase 1):

Sociodemographic variables and work experience: sex; age; years of professional experience; years of experience in PHC; years performing fundus photography tests. 

Fundus photograph assessment and screening variables: screening results of each image (normal, non-normal, or invalid image); lesions observed in each image (hard exudates, cotton wool spots, haemorrhages, or microaneurysms); presence/degree of DR (invalid image, no DR, mild DR, moderate DR, or severe DR).

Variables relating to knowledge of DR. Other secondary, partial, and global variables related to DR knowledge levels were generated based on the previous variables. These variables are the sum of matching answers from each of the three different assessment aspects (screening, lesions observed, and presence/degree of DR): number of matches for each image (between 0 and 3); number of overall matches in aspect 1: screening (between 0 and 20); number of overall matches in aspect 2: lesions observed (between 0 and 20); number of overall matches in aspect 3: presence/degree of DR (between 0 and 20). In addition, one final variable was generated—the sum of the three previous variables—the number of overall matches (between 0 and 60).

Patient variables (phase 2):

Sociodemographic variables: sex and age.

Clinical variables and health habits: years since DM was diagnosed; pharmacological treatment; HbA1C values in the past year; hypertension (HT); dyslipidaemia; physical exercise habits; body mass index (BMI).

Fundus photography screening result: assessed as normal or not normal by both the PHC nurse and the ophthalmologist at the referral hospital.

The result of the fundus photograph was expressed as the suspected presence of DR and degree of damage (no DR, mild DR, moderate DR, or severe DR) both by the nurse and the ophthalmologist.

### 2.5. Data Analysis 

Nominal variables were summarised using the absolute frequency of their categories, while scalar variables were summarised using means and standard deviations (SDs) or medians and 5th and 95th percentiles (Pc5-Pc95) depending on the normality of their distribution. Bivariate analysis was performed using the relevant tests to explore associations and differences depending on the type of variables involved and the normality of the distribution of scalar variables. As a result, Pearson or Spearman correlation coefficients, Student’s *t*-tests or Mann–Whitney’s *U*-tests, and chi-squared tests were used. Sensitivity, specificity, and predictive values were also calculated. Inter-professional reliability analysis (nurses and ophthalmologists) for screening and assessment of fundus photographs was performed using the raw agreement of the records and the unweighted Cohen’s kappa coefficient. The Landis and Koch criteria [20] were used as a reference to assess the strength of agreement of the kappa coefficients obtained: slight (0.01–0.20); fair (0.21–0.40); moderate (0.41–0.60); substantial (0.61–0.80); almost perfect (0.81–1.00). All tests were two-tailed and performed at an alpha significance level of <0.050 using SPSS© (v.25.0.) software from IBM (IBM corporation, Chicago, IL, USA).

## 3. Results

### 3.1. Characteristics of Participating Nurses and Their DR Knowledge Level

In this first phase, 70 of the 165 nurses who were eligible for inclusion agreed to participate in the study. A total of 71.4% were women and the mean age was 44.5 (7.9) years. The mean number of years of professional experience was 20.2 (8.5). In terms of experience in PHC and fundus photography, the median number of years was 13.0 (1.0–29.4) and 1.5 (1.0–13.4), respectively. Fifty percent of participants had less than two years of experience performing fundus photography.

Table 1 shows the percentages of matches for each of the three aspects evaluated in each image, as well as the correct results for all of them and the total percentage of matches for each image (between 0 and 3). The highest match rates were observed in the screening results (normal, non-normal, or invalid image). On average, 68.4% of the sample obtained 2–3 matches per image.

The mean scores (between 0 and 20) obtained by the nurse regarding the overall scores for each of the three aspects assessed (screening, observed lesions, and presence/degree of DR) were 18.2 (1.6), 9.9 (2.2), and 10.8 (3.0), respectively. The overall mean total score, i.e., the sum of the three aspects assessed (0–60), was 38.9 (4.2).

No significant differences were identified between sex and level of knowledge: overall, the mean total score between women and men was 39.5 (4.0) matching answers versus 37.4 (4.4) matching answers, respectively (*p* = 0.054). No significant associations were identified between the overall mean total score and age (Spearman’s rho correlation coefficient = 0.164; *p* = 0.176); years of experience (Spearman’s rho = 0.100; *p* = 0.410); length of time taking fundus photographs (Spearman’s rho = 0.226; *p* = 0.060). However, a significant direct association was found between the number of years working in PHC and the number of overall image screening matches (normal, non-normal, or invalid images), with a Spearman’s rho correlation coefficient of 0.275, *p* = 0.021.

### 3.2. Characteristics of Patients with Diabetes

Phase 2 of the study included fundus photographs of 100 individuals with type 2 DM under the care of nine nurses from five ZBSs participating in phase 1, who agreed to be included in this new phase of the research. The images were randomly selected from EHRs between August and September 2022. Regarding patient characteristics, 55% were male and the mean age was 66.2 (11.0) years. The median number of years since the onset of DM was 5 (1–16.0). Fifty-seven percent of the patients had been living with DM for 5 years or less, 19% for 6 to 10 years, and 24% for more than 10 years. Ninety-two percent were taking oral antidiabetic drugs (OADs) and 41% were taking insulin. The mean HbA1C value among patients with DM was 6.9 (1.1)%, with 59.8% of them having values below 7%, and 86.6% having values below 8%. Additionally, 74.7% had high blood pressure and 71% had dyslipidaemia. In terms of physical exercise habits, 24.2% were active, 48.5% were partially active, and 27.3% were inactive, as reported on their EHRs. The median BMI of the sample was 31.2 (22.5–41.0): 14.6% were below 25 (ideal weight); 24.7% were between 25 and 29.9 (overweight); 44.9% were between 30 and 34.9 (obese I); 7.9% were between 35 and 39.9 (obese II); 7.9% were 40 or above (obese III, morbid obesity).

### 3.3. Level of Agreement between Nurses and Ophthalmologists

The results of fundus photograph screenings were that 59% of cases were classed as non-normal by nurses and 62% by ophthalmologists. Regarding the suspected presence of DR and the levels of lesions, nurses identified mild DR in 45% of cases and moderate DR in 12% of cases, while ophthalmologists diagnosed mild DR in 48% of cases and moderate DR in 11% of cases.

The overall raw agreement between nurses and ophthalmologists in the screening of fundus photograph results was 75%, with a Cohen’s kappa value of 0.477 (*p* < 0.001) (Table 2). The nurses’ ability to recognise non-normal images through fundus photography (test sensitivity) was 77%, while their ability to detect a normal image (test specificity) was 71%, with positive predictive values (PPVs) of 81% and negative predictive values (NPVs) of 66%. 

In addition, the agreement between both professional profiles on the presence of DR and the severity of lesions was 56%, with a Cohen’s kappa value of 0.260 (*p* = 0.001) (Table 3).

When dividing the sample into two groups—first by years since diagnosis of DM (5 years or less and more than five years since diagnosis of DM) and then by HbA1C results (below 7% and greater than or equal to 7%)—better results for overall agreement and kappa coefficients were found for some of the values (Table 4).

The overall raw agreement—on screening fundus photography results for patients who had been living with the disease for five years or less—was 75.5%, with a sensitivity of 77%, a specificity of 74%, a PPV of 77%, an NPV of 74%, and a Cohen’s kappa value of 0.507 (*p* < 0.001) (Table 4). For those with HbA1C values below 7%, the overall raw agreement was 77.6%, with a sensitivity of 80%, a specificity of 75%, a PPV of 77%, NPV of 78%, and a Cohen’s kappa values of 0.551 (*p* < 0.001). Similarly, in these cases, the agreement between nurses and ophthalmologists regarding the presence of DR and the severity of lesions was 59.7% and 60.3%, with kappa coefficients of 0.315 (*p* = 0.003) and 0.299 (*p* = 0.006), respectively.

## 4. Discussion

### 4.1. Level of Knowledge

Care provision and health promotion are fundamental aspects of every healthcare system. For care provision to be effective, professionals must keep up to date with the evolving knowledge of their disciplines. Continued professional development is a tool that is known to help healthcare professionals adapt to the ongoing changes and demands of society [21], while also being considered both a right and an obligation enshrined in the professional codes of ethics and Law 44/2003 of 21 November, regulating health professions in Spain [22]. When screening for DR, evidence recommends specific formal training for the professionals involved in both taking and interpreting photographs [23]. One of the most important practical aspects in the assessment of fundus photography nurse training is its impact on skills acquisition, defined as the measurement of the outcomes of training activities performed over a given period [24,25]. Therefore, in the first part of the study, we sought to establish the DR knowledge level as a criterion for the quality of the skills acquired. To this end, we selected three indicators that can objectively measure knowledge of fundus photography: screening (normal, non-normal, or invalid image), observed lesions (microaneurysms, haemorrhages, hard exudates, and cotton wool spots), and the presence and degree of DR (no DR, mild DR, moderate DR, severe DR).

The last two indicators (observed lesions and suspected diagnosis) yielded a 50% match rate. The training programme focuses on operating the fundus camera to obtain images of high diagnostic quality. Having to learn to handle a technology that differs greatly from what nurses are used to means that they must initially focus their attention and efforts on the technical aspects. If nurses are not able to obtain good-quality images, these will not be sent by the telemedicine system for evaluation, and if they are sent, they will be sent back to be repeated. Furthermore, in current clinical pathways, lesion description and diagnostic classification in PHC are tasks assigned to general practitioners. Therefore, it seems reasonable that nurses do not prioritise this type of knowledge, placing more emphasis on identifying the image as normal or non-normal for prompt referral [12]. Although half of the nurse sample had been performing fundus photography for less than two years, no significant differences were identified between the number of years performing fundus photography and level of knowledge. However, a significant association was found between the nurse’s experience in PHC and the number of overall matches in screening images.

On the other hand, when the nurses in our sample screened by classifying the images as normal, non-normal, or invalid, the match rate was 90%, with a correlation being found between better screening, and more years of experience in PHC. This high match rate and the association found between better screening skills and work experience may be linked to the fact that part of their routine work in the clinic is diabetes-related eye health education, for which it is important to know whether or not the patient’s image is within the normality range, allowing for personalised diabetes education to be provided. In addition, the fact that nurses are responsible for obtaining good-quality imaging is likely to encourage them to adopt a more participatory role in identifying non-normal images, resulting in a high match rate [26,27].

### 4.2. Agreement between Nurses and Ophthalmologists

This study reports a moderate level of overall agreement between nurses and ophthalmologists, falling short of the recommended kappa agreement levels required for DR screening, which are around 80%, as well as the necessary ~90% sensitivity and specificity values [28,29,30]. This study obtained moderate agreement values between nurses and ophthalmologists in terms of overall raw agreement in screening (Table 2), especially in patients with less disease progression and better disease control (Table 4). However, these results are not optimal, which points to the need to reconsider improving the education and training of nurses who perform fundus photography on diabetic patients in the health area in question. Discrepancies when interpreting fundus photographs are neither new in our healthcare context nor confined solely to a nursing assessment of fundus photograph results. A study by Plasencia et al. in 2013 reported a high degree of disagreement in the diagnoses made by general practitioners and ophthalmologists. The authors argued that this disagreement was probably due to general practitioners being aware that they act as first screeners and are thus more sensitive to any suspicious lesions [31], a hypothesis that could be extrapolated to nurses. Therefore, as can be observed in the results of our research in terms of higher observed sensitivity and better positive predictive values, participating nurses’ assessment of the fundus photography results is particularly valuable to screening.

The agreement values obtained in this study regarding the presence of DR and the severity of this condition are acceptable and significant, but at the same time too low for what would be expected in terms of appropriate diagnostic agreement. This is understandable, and perhaps logical, given that nurses, in our opinion, are generally not specialised enough to identify the specific extent of these lesions. It is well known that practitioners who are properly trained and who have acquired the necessary experience through the practical reading of images over a certain period of time can reach the desired values when it comes to identifying lesions associated with suspected DR [16,17,18]. As such, the training programme that the nurses participating in this study receive focuses primarily on the eye fundus of diabetic patients, the technical equipment to be used for imaging, and the logistical aspects of this type of consultation in a PHC facility. It goes without saying that the recommended educational models for training non-ophthalmologists in interpreting DR images include exclusive training focused on the assessment of many images. This should also be spread out over time and supplemented with feedback from experienced professionals [27,29]. In addition, this training should be updated over time with periodic re-accreditation programmes to maintain competence and can be complemented using clinical simulators [32]. Therefore, in order to improve the diagnostic agreement between nurses and ophthalmologists arising from our research, it is recommended that new measures be implemented within our context aimed at establishing a training model based on the afore-mentioned characteristics, which would increase the number of image assessments with the direct help of expert professionals and the use of clinical simulators.

On the other hand, in our study, the raw agreement and kappa coefficient values increased significantly when assessing images of individuals who had been living with DM for shorter periods of time and with better metabolic control (Table 4). It is possible that the fundus images of these patients, with few or no DR lesions, are mostly normal and, consequently, even with little specific training, nurses may be able to identify them with greater accuracy than in images from individuals who have had the disease for a longer period or with poorer metabolic control. 

This study features several limitations. Firstly, our results are not comparable with other healthcare areas where there are no fundus photography training programmes for nurses with similar characteristics to the one implemented in our setting. Secondly, the participant selection method may involve a notable bias, given that, among the 165 nurses trained in our health area to perform fundus photography, the 70 who finally participated did so voluntarily. This may be indicative of their motivation to do well in an area of expertise that they feel more comfortable performing than the nurses who chose not to participate. Finally, the nine nurses participating in the second phase could have been even better trained and more willing to participate. Nevertheless, the results point to a need for further training and competence development in the identification and specification of fundus lesions in patients with diabetes.

## 5. Conclusions

The average nurse participating in this research is female, with 20 years of professional experience and 13 years of experience in PHC. Half of them have less than two years experience of performing outpatient fundus photography on diabetic patients. They possess a high level of knowledge in terms of how the screening test is performed, but there is room for improvement regarding the types of lesions observed and diagnostic suspicion. Their overall raw agreement with ophthalmologists on screening was 75%, with a moderate degree of agreement and similar values when the patient has been living with the disease for a short time or has good control of the disease. The agreement values for DR severity were acceptable but need to be improved. Therefore, in light of these results, further training measures should be implemented to broaden nurses’ knowledge in this field.

## Figures and Tables

**Table 1:** Percentages of partial and total matches for each aspect of fundus photographs assessed by nurses (*n* = 70).

Image	Screening Result *	Matches (%)	Lesions Observed **	Matches (%)	Suspected Diagnosis ***	Matches (%)	Distribution (%) in Number of Matches per Image			
							0	1	2	3
1	nn	100	haem	57.1	midr	72.9	0	11.4	47.1	41.4
2	nn	87.1	mic	72.9	nodr	18.6	0	25.7	70.0	4.3
3	nn	94.3	haem	58.6	midr	61.4	5.7	11.4	45.7	37.1
4	nn	87.1	haem, mic	7.1	midr	32.9	12.9	51.4	31.4	4.3
5	nn	100	haem	34.3	modr	57.1	0	28.6	51.4	20.0
6	n	91.4	nl	91.4	nodr	92.9	5.7	2.9	1.4	90.0
7	nn	85.7	mic	57.1	nodr	15.7	2.9	40.0	52.9	4.3
8	nn	100	hex, haem	14.3	midr	44.3	0	45.7	50.0	4.3
9	n	92.9	nl	92.9	nodr	91.4	7.1	0	1.4	91.4
10	nn	88.6	hex, mic	24.3	midr	68.6	11.4	17.1	50.0	21.4
11	iv	78.6	nl	70.0	iv	85.7	10.0	14.3	7.1	68.6
12	nn	82.9	cws	71.4	nodr	25.7	4.3	24.3	58.6	12.9
13	nn	98.6	mic	32.9	midr	38.6	0	48.6	32.9	18.6
14	nn	78.6	nl	17.1	nodr	24.3	1.4	77.1	21.4	0
15	nn	98.6	mic	18.6	midr	38.6	1.4	54.3	31.4	12.9
16	nn	97.1	haem, mic	50.0	modr	71.4	1.4	12.9	51.4	34.3
17	nn	85.7	mic	68.6	nodr	21.4	1.4	28.6	62.9	7.1
18	nn	68.6	mic	47.1	modr	51.4	31.4	10.0	18.6	40.0
19	nn	98.6	cws, mic	7.1	modr	65.7	0	30.0	68.6	1.4
20	iv	94.3	nl	98.6	iv	97.1	1.4	0	5.7	92.9
Average percentage of matches (%)		90.4		49.6		53.8	4.9	26.7	38.0	30.4

* n: normal image; nn: non-normal image; iv: invalid image. ** nl: no lesions; hex: hard exudates; cws: cotton wool spots; haem: haemorrhages; mic: microaneurysms. *** iv: invalid image; nodr: no diabetic retinopathy; midr: mild diabetic retinopathy; modr: moderate diabetic retinopathy.

**Table 2:** Agreement between nurses and ophthalmologists on fundus imaging screening results in patients with diabetes (*n* = 100).

		Result of Fundus Photograph Assessment by Ophthalmologists (%)		Total %
		Non-Normal Image	Normal Image	
Result of fundus photograph assessment by nurses (%)	Non-normal image	48	11	59
	Normal image	14	27	41
Total %		62	38	100

**Table 3:** Agreement between nurses and ophthalmologists in the identification and grading of diabetic retinopathy after assessment of fundus photographs (*n* = 100).

		Result of Fundus Photograph Assessment by Ophthalmologists (%)			Total %
		No DR *	Mild DR	Moderate DR	
Result of fundus photograph assessment by nurses (%)	No DR	28	14	1	43
	Mild DR	13	25	7	45
	Moderate DR	0	9	3	12
Total %		41	48	11	100

* DR: Diabetic retinopathy.

**Table 4:** Agreement between nurses and ophthalmologists on the results of fundus photograph screening and on the identification and degree of diabetic retinopathy by ‘years since diabetes mellitus onset’ and by ‘monitoring values’.

**By Years since Diabetes Mellitus Onset**											
**5 years or less since onset**							**More than 5 years since onset**				
		Result of fundus photograph assessment by ophthalmologists % (*n*)					Result of fundus photograph assessment by ophthalmologists % (*n*)				
		Non-normal image		Normal image		Total % (*n*)	Non-normal image		Normal image		Total % (*n*)
Result of fundus photograph assessment by nurses % (*n*)	Non-normal image	40.4 (23)		12.3 (7)		52.6(30)	58.1 (25)		9.3 (4)		67.4 (29)
	Normal image	12.3 (7)		35.1 (20)		47.4 (27)	16.3 (7)		16.3 (7)		32.6 (14)
	Total % (*n*)	52.6 (30)		47.4 (27)		100 (57)	74.4 (32)		25.6 (11)		100 (43)
S = 77%; E = 74%; PPV = 77%; NPV = 74%; kappa = 0.507; *p* < 0.001							S = 78%; E = 64%; PPV = 86%; NPV = 50%; kappa = 0.383; *p* = 0.011				
		No DR	Mild DR		Moderate DR	Total % (*n*)	No DR	Mild DR		Moderate DR	Total % (*n*)
Result of fundus photograph assessment by nurses % (*n*)	No DR	35.1 (20)	8.8 (5)		0 (0)	43.9 (25)	18.6 (8)	20.9 (9)		2.3 (1)	41.9 (18)
	Mild DR	14.0 (8)	22.8 (13)		8.8 (5)	45.6 (26)	11.6 (5)	27.9 (12)		4.7 (2)	44.2 (19)
	Moderate DR	0 (0)	8.8 (5)		1.8 (1)	10.5 (6)	0 (0)	9.3 (4)		4.7 (2)	14.0 (6)
	Total % (*n*)	49.1 (28)	40.4 (23)		10.5 (6)	100 (57)	30.2 (13)	58.1 (25)		11.6 (5)	100 (43)
kappa = 0.315; *p* = 0.003							kappa = 0.186; *p* = 0.102				
**By HbA1C Results**											
**Values below 7%**							**Values greater than or equal to 7%**				
		Result of fundus photograph assessment by ophthalmologists % (*n*)					Result of fundus photograph assessment by ophthalmologists % (*n*)				
		Non-normal image		Normal image		Total % (*n*)	Non-normal image		Normal image		Total % (*n*)
Result of fundus photograph assessment by nurses % (*n*)	Non-normal image	41.4 (24)		12.1 (7)		53.5 (31)	59.0 (23)		7.7 (3)		66.7 (26)
	Normal image	10.3 (6)		36.2 (21)		46.5 (27)	20.5 (8)		12.8 (5)		33.3 (13)
	Total % (*n*)	51.7 (30)		48.3 (28)		100 (58)	79.5 (31)		20.5 (8)		100 (39)
S = 80%; E = 75%; PPV = 77%; NPV = 78%; kappa = 0.551; *p* < 0.001							S = 38%; E = 33%; PPV = 10%; NPV = 72%; kappa = 0.298; *p* = 0.050				
		No DR	Mild DR		Moderate DR	Total % (*n*)	No DR	Mild DR		Moderate DR	Total % (*n*)
Result of fundus photograph assessment by nurses % (*n*)	No DR	37.9 (22)	8.6 (5)		1.7 (1)	48.3 (28)	12.8 (5)	23.1 (9)		0 (0)	35.9 (14)
	Mild DR	15.5 (9)	22.4 (13)		3.4 (2)	41.4 (24)	7.7 (3)	28.2 (11)		12.8 (5)	48.7 (19)
	Moderate DR	0 (0)	10.3 (6)		0 (0)	10.3 (6)	0 (0)	7.7 (3)		7.7 (3)	15.4 (6)
	Total % (*n*)	53.4 (31)	41.4 (24)		5.2 (3)	100 (58)	20.5 (8)	59.0 (23)		20.5 (8)	100 (39)
kappa = 0.299; *p* = 0.006							kappa = 0.156; *p* = 0.168				

DR: Diabetic retinopathy; HbA1C: glycosylated haemoglobin; S: sensitivity, E: specificity; PPV: positive predictive value; NPV: negative predictive value.

## Data Availability

The data presented in this study are available upon request from the corresponding author. The data are not publicly available due to privacy/ethical restrictions.

## Public Involvement Statement

No public involvement in any aspect of this research.

## Guidelines and Standards Statement

This manuscript was drafted against the STROBE guidelines for observational studies [33].
